# Why is hitting A&E time targets so hard?: using Nudge theory and modelling to improve response times

**DOI:** 10.1192/bjo.2021.595

**Published:** 2021-06-18

**Authors:** Kaj Svedberg

**Affiliations:** City and hackney Centre for Mental Health

## Abstract

**Aims:**

To improve the one hour response times to referrals made to psychiatric Liaison in A&E without adding or changing available resources.

**Method:**

Response time data of referrals made to the Homerton University Hospital psychiatric liaison service was collected dating back from August 2016 to October 2019 (n = 10225).

A nudge was introduced in the form of a large display showing referrals arriving in real time in the staff office.

Data was then collected over a period of 5 weeks (n = 436) to measure if any change had occurred in response times.

**Result:**

Response times appear to follow a Poisson like distribution curve. The average referral was responded to within 6 minutes (n = 1577) prior to the nudge, and 6 minutes (n = 88) after. Prior to the nudge the 95% referral envelope fell within 134 minutes (n = 9728) and was 122 minutes (n = 414) after the intervention. Significant statistical difference is observed upon considering response in the first 240 minutes.

**Conclusion:**

Nudge interventions could be a useful resource-sparing method to improve services. The average referral to the HUH liaison team was quickly responded to within 6 minutes and yet hitting the 1 hour 95% target appears ever-elusive. Hitting targets of 95% responses within 1 hour may prove very difficult if we are not considering natural distributions, such as Poisson, occuring in the backgroung which ultimately may require a change in approaches to how we set performance targets.

